# How Does It Feel to Be a Pathologist in Turkey? Results of a Survey on Job Satisfaction and Perception of Pathology

**DOI:** 10.5146/tjpath.2020.01513

**Published:** 2021-01-15

**Authors:** Burcin Pehlivanoglu, Hur Hassoy, Gulen Gul, Umut Aykutlu, Basak Doganavsargil

**Affiliations:** Department of Pathology, Adiyaman University Training and Research Hospital, Adıyaman, Turkey; Department of Public Health Ege University, Faculty of Medicine, Izmir, Turkey; Department of Pathology, Tepecik Training and Research Hospital, Izmir, Turkey; Department of Pathology, Ege University, Faculty of Medicine, Izmir, Turkey

**Keywords:** Surgical pathology, Pathologists, Job satisfaction, Perspective, Minnesota satisfaction questionnaire

## Abstract

***Objective:*** Job satisfaction affects productivity and professional performance in many aspects; however, there is limited data regarding pathologists’ job satisfaction. Hence, in this study, we aimed to evaluate surgical pathologists’ job satisfaction in Turkey.

***Materials and Methods: *** We conducted a 59-item web-based survey questioning respondents’ institutional background, history of training, continuing education status/research activities, physical conditions, professional well-being, and job satisfaction level. Likert-type and open/close ended questions were asked and scored. The participants were also asked to complete the Minnesota Satisfaction Questionnaire-Short Form.

***Results: *** Of the 321 respondents, 75% were female, the median age was 41 years (range 28-71 years), experience as a pathologist ranged between 0.12 and 44 years (mean 11.4±9.16 years). Academic pathologists, senior pathologists with ≥20 years of experience, and pathologists working at large institutions and living in developed cities expressed better physical conditions, higher satisfaction with working conditions and, therefore, higher overall job satisfaction (p<0.05). 98% agreed that pathologists have a critical impact on patient management; however, the majority (>80%) thought that patients barely know what pathologists do and other physicians rarely understand the difficulty and limitations in pathology practice. 82% were happy to have chosen pathology but 45% reported to experience the feeling of being “burnt out”.

***Conclusions: *** Our findings suggest that younger pathologists are less satisfied with their jobs and a surgical pathologist’s job satisfaction increases with the physical and technical quality of the pathology laboratory/institution, and years of experience. Pathologists seem to be aware of their important role in patient management although they think that pathology remains “invisible” to many physicians and patients.

## INTRODUCTION

Every cell has a history (1) and pathologists try to reveal this history, often amazed by the view they see through the microscope. However, pathology is not just microscopic examination, and most of the time, microscopy is just a small part of a pathologist’s daily routine and it is quite possible that his/her professional enthusiasm starts to fade away and disappear after several years of multitasking.

Locke has described job satisfaction as a pleasurable or positive emotional state resulting from one’s job experiences and claimed that it is determined by the discrepancy between what one wants and gets in a job (2). While job satisfaction directly affects one’s motivation and workplace performance, it would be unrealistic to consider that every individual has the same expectations in professional life since not every person is at the same level according to Maslow’s hierarchy of needs (3). However, it is also true that a job must offer some basic qualities at least to fulfill certain needs of an individual.

To the best of our knowledge, there are only a few studies on the job satisfaction of surgical pathologists (4–6), some of which focus on the residency period (7,8). In this study, we aimed to evaluate the job satisfaction levels of pathologists in Turkey and their perspective on pathology.

## MATERIAL and METHODS

### Survey Design and Data Collection

The study protocol was approved by the institutional ethics committee(Approval no: 18-5.1/4). We conducted a 60-item survey questioning the respondents’ 1) institutional background and history of training, 2) workplace physical conditions (WPC), 3) continuing education status/research activities, 4) professional well-being (PWB; can be defined as positive perceptions and favorable conditions at work that meet occupational needs (9,10), or having a “high-quality” work life while maintaining the work/life balance) and job satisfaction, and 5) perception of pathology. The survey was delivered via a web-based link and the questions were based on the authors’ observations and self-experience as well as previous job satisfaction surveys (7,11) and Ford’s study on the specialty choices of medical students (12). Different question types including Likert-type questions, yes/no questions, open/close ended questions were used. A 5-point score was used for Likert-type questions: 1: Strongly disagree, 2: Somewhat disagree, 3: Neutral, 4: Somewhat agree, and 5: Strongly agree. The participants were also asked to complete the Minnesota Satisfaction Questionnaire-Short Form (MSQ)(13) in order to compare the results to a validated job satisfaction questionnaire.

### Sorting Data

Respondents were grouped based on their age, gender, experience as a pathologist, the size and experience of their laboratories, and the type of the hospital they worked/trained at. Institutions with an actively working pathology laboratory for >20 years were considered “experienced”. The experience of the respondents was also grouped: 1) 0-2 years, 2) 3-10 years, 3) 11-19 years and 4) **≥ **20 years. Institutions with a total biopsy number of >40.000 were considered “large” (1-20.000 biopsies: small; 20.001-40.000: medium-sized). The provincial development level, i.e., the level of socio-economic development in a residential area/city, was grouped per the data provided by Ministry of Development (14). Mean scores were calculated for Likert-type questions (including MSQ). A cut-off level of 3.5 points was determined as the mean dimension score based on previous studies (15). Likert-type questions and the answer scores (between 1 and 5 points) were also directly correlated with other variables while evaluating job satisfaction and perception of pathology. The outcome variables were converted into dichotomous variables for statistical analyses. Similar answers for partial/complete open-ended questions were grouped in the same category for homogeneity.

Based on our initial findings, we divided the respondents into four groups to compare the variables: 1) non-academic junior, 2) academic junior, 3) non-academic senior, and 4) academic senior. 40 years of age was determined as the cut-off for seniority (**≥**40 years) (16). Pathologists with an academic title were included in the academic groups. All parameters were also compared between the geographical regions of Turkey.

### Statistical Analysis

Statistical analysis was performed using the SPSS version 20.0 (SPSS Inc. Chicago, IL) software. Mean and/or median values were presented as mean±standard deviation (SD) and/or median (interquartile range [IQR]). Frequencies were compared via the chi-square test. Mean scores were calculated for Likert-type questions and compared using non-parametric tests based on the results of the homogeneity analyses demonstrating that the data did not show a normal distribution. Two independent groups were compared using the Mann-Whitney U test, as the Kruskal-Wallis test was used to compare >2 independent groups. The Dunn test was performed as a post-hoc test to further analyze the results of the Kruskal-Wallis test and to better demonstrate the significant differences between the subgroups. A value of p<0.05 (two-sided) was considered statistically significant.

## RESULTS

### Institutional Background

A total of 321 pathologists from 73 cities completed the survey. The response rate (calculated based on the total number of pathologists in Turkey (17)) was 24%. The majority (75%; n=242) were female with a median age of 41 years (IQR: 15; mean 42±8.82 years). The respondents’ experience as a pathologist differed widely (range: 0.12 years to 44 years; mean 11.4±9.16 years). More than half (56%; n=181) worked at medium-sized or large institutions and 36% (n=116) had academic titles (Table 1). However, 35% (n=113) (mainly pathologists <40 years old, p=0.023) stated that the institution they worked at was not their primary choice. The majority (89%; n=286) worked at a previously established pathology laboratory.

**Table 1:** Demographic characteristics and institutional background of the respondents.

**Age**	Median 41 years (IQR: 15)
**Gender**	Female 75% (n=242), Male 25% (n=79)
**Duration of working as a pathologist**	Mean: 11.4±0.48 years (Range 0.12-44 years)
**Time since the establishment of the pathology laboratory** ** <2 years** ** 2-10 years** ** 11-20 years** ** >20 years**	3% (n=11) 22% (n=72) 29% (n=92) 46% (n=146)
**Number of respondents per institution type** ** Private hospital /laboratory** ** State hospital** ** Training and research hospital** ** University hospital**	8% (n=26) 35% (n=112) 28% (n=89) 29% (n=94)
**Number of biopsies per year (including cytology specimens)** ** 1-20.000** ** 20.001-40.000** ** >40.000**	44% (n=141) 40% (n=127) 17% (n=53)
**Number of cervicovaginal smear samples per year** ** 1-1.500** ** 1.1501-4.000** ** >4000**	21% (n=66) 30% (n=95) 50% (n=160)
**Number of pathologists per institution** ** 1-5** ** 6-10** ** 11-15** ** >15**	45% (n=144) 33% (n=197) 12% (n=39) 10% (n=31)
**Number of pathologists with an academic title** ** Assistant professor** ** Associate professor** ** Professor**	9% (n=29) 11% (n=36) 16% (n=51)
**Number of technicians per institution** ** 1-5** ** 6-10** ** 11-15** ** >15**	34% (n=109) 39% (n=126) 13% (n=41) 14% (n=45)
**Number of administrative staff per institution** ** 0** ** 1-5** ** 6-10** ** 11-15** ** >15**	3% (n=11) 68% (n=219) 23% (n=74) 3% (n=9) 3% (n=8)

### History of Training

Pathology was not the first specialty choice of 70% (n=225), and 66% (n=213) did not have adequate information about routine pathology practice when they graduated from medical school. Most of the participants (73%; n=235) had been trained at a university hospital; however, barely more than half (52%; n=167) stated that they were confident about their knowledge and skills on pathology when they completed their residency period. Molecular pathology, administration/management strategies, and laboratory techniques seemed to be the weakest aspects of the residency programs (Supplement 1). Moreover, 21% (n=67) reported a fear of responsibility/lack of confidence to sign-out cases when they first started practicing, as 21% (n=66) reported inexperience/lack of knowledge on several topics of pathology, 20% (n=63) inexperience in laboratory management, and 14% (n=46) lack of knowledge on laboratory techniques. Remarkably, 11 of the 63 respondents who reported inexperience in laboratory management had to establish the laboratory themselves as they were assigned to hospitals without a pathology laboratory while 6% (n=18) complained about worse laboratory conditions than they were used to.

The respondents that had been trained at a university hospital were more satisfied with their training (p<0.001). Interestingly, male participants were more satisfied with their training (p=0.031). Training satisfaction scores of academic and senior pathologists were significantly higher (p<0.001).

### Workplace Physical Conditions (WPC)

While almost two thirds (65%; n=208) appeared to be satisfied with their routine tissue processing and staining procedures, barely half (49%; n=157) thought that the technical and physical background of their laboratory was satisfactory despite the presence of automated tissue processing and/or staining systems and experienced technical staff in many laboratories (Supplement 2). The vast majority (88%; n=285) stated that turn-around times for sign-out had been determined and written in the test guidelines. Incomplete clinical information was considered the most significant factor interfering with turnaround time, followed by ancillary tests such as immunohistochemistry etc. (Supplement 3). Unfortunately, 22% (n=70) reported a verbal disagreement with a patient or patient’s next of kin, and 2% (n=6) reported physical abuse mainly regarding turn-around time, payments for the ancillary tests, or a diagnosis of malignancy.

Academic pathologists and senior pathologists with ≥20 years of experience working at larger laboratories were significantly more satisfied with their WPC (p<0.001 and p=0.019). Respondents who worked at large or experienced institutions also had better technical background and physical condition satisfaction levels, and they stated to be working with more experienced staff/technicians (p<0.001). WPC satisfaction level, technical background scores, and the quality of the staff/technicians were also significantly associated with the level of provincial development of the city the respondents lived in (p<0.001).

### Continuing Education Status/Research Activities

#### Getting a second opinion (consultation)

Of the 321 respondents, 65% (n=210) reported that they did not subspecialize on certain fields. While the majority (93%; n=298) consulted the cases intradepartmentally, hematolymphoid pathology (33%, n=105) was the most frequent subspecialty creating a need for a second opinion, followed by dermatopathology including tumoral lesions (16%, n=51) and soft tissue/bone pathology (13%, n=42).

#### Attendance at scientific meetings

Almost half (47%; n=151) stated that no clinicopathologic meeting was being held at their institutions. Only 5% (n=15) reported that they regularly attend monthly pathology meetings/courses held by regional pathology societies, while the majority (41%; n=130) reported this number as once or twice a year. The number of respondents who annually attended national and international pathology meetings was low (28% (n=91) for the national pathology meeting and 6% (n=18) for international pathology meetings). The most important factors that determined the decision to attend these meetings were the quality of the scientific content (85%; n=273) and financial status/support (76%; n=244).

#### Research activities and publication ethics

Almost two thirds had contributed to at least one original research article and/or case report, also in cooperation with other specialties (60%; n=193, 66%; n=212, respectively). 28% (n=90) claimed that their cases were included in studies without their knowledge, or they were not listed as a co-author (6%; n=18) although they fulfilled authorship criteria by providing rare diagnoses, micro-photos, performing further histopathological examination, etc. Interestingly, another pathologist had been listed as a co-author instead of the person who initially provided the data in 3 of these incidents.

### Professional Well-being (PWB) and Overall Job Satisfaction

**Figure 1:**
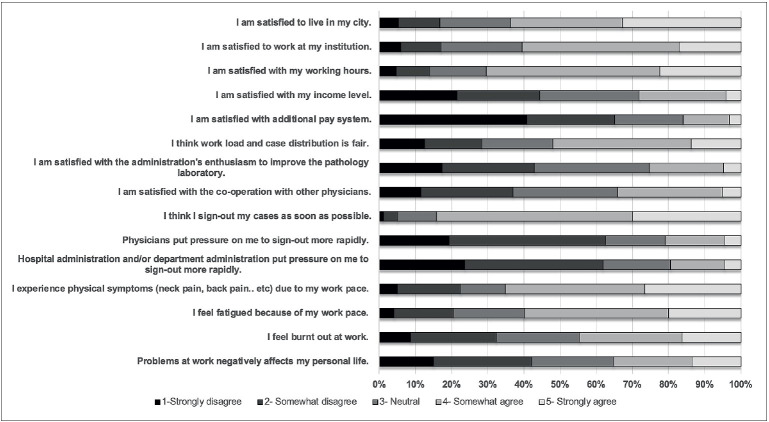
Answers to Likert-type questions on professional well-being and job satisfaction.

More than half of the respondents were satisfied with the city they lived in (64%; n=204), and the institution they worked at (60%; n=194) (Figure 1). However, almost half (45%; n=143) reported experiencing the feeling of burn out, regardless of their seniority and academic status (p>0.05). 65% (n=209) reported having physical problems such as back pain or neck pain and 59% (n=192) described fatigue due to excessive working. In addition, 52% (n=167) thought that case load/distribution in their department was not fair and 28% (n=89) stated that they experienced conflicts with their colleagues (Supplement 4). The most common reasons for the conflicts were personal disagreements (64/89), case distribution (30/89), subspecialization (23/89), monthly income (22/89), and differences in scientific/diagnostic approach (20/89).

Respondents who worked at large or experienced institutions and academic pathologists expressed higher satisfaction with working conditions, and the PWB score was also significantly correlated with the level of provincial development of the city the respondents lived in (p<0.001). Pathologists from smaller institutions reported significantly less conflict with their colleagues (p=0.007). Female respondents were more satisfied with their working conditions and reported having better relationship with colleagues (p<0.05). Finally, the majority were not satisfied with their wage and salary supplements (76%; 80% per MSQ scores) (Figure 1, Supplement 5) and 64 respondents (20%) also described inadequate financial compensation as a discouraging factor.

### Perception of pathology

**Figure 2:**
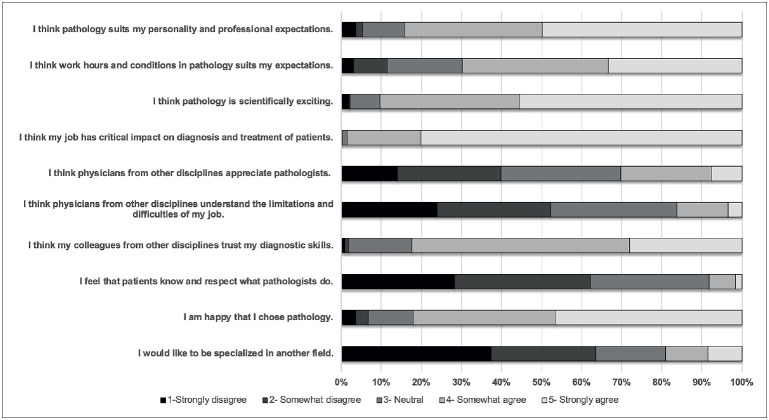
Answers to the questions about the perception of pathology.

Overall, 82% (n=263) of the participants were happy to have chosen pathology (Figure 2), and interestingly happiness was significantly associated with the level of provincial development of the city they lived in (p=0.024). The most common factors that made the respondents happy were flexible working hours/conditions (34%), to have a high impact on patient management (18%), and to get and to make the diagnosis (14%) (Supplement 6).

Almost all (98.4%; n=316) agreed that pathologists have a critical impact on patient management, but 92% (n=295) thought that patients barely know about pathologists. The ratio of the respondents who agreed that other physicians understand the difficulty and limitations in pathology practice was only 16% (n=52) (Figure 2), and only 25% (n=85) were satisfied with the hospital administration’s enthusiasm to improve the pathology laboratory (Figure 1). The perception of pathology of the respondents from large and experienced institutions was more favorable (p=0.021 and 0.015, respectively).

### Minnesota Satisfaction Questionnaire

Academic pathologists and senior pathologists with **≥**20 years of experience had significantly higher scores of MSQ (p<0.001)(Supplement 5). The mean MSQ score was also significantly associated with the mean training satisfaction score (p=0.29), WPC satisfaction score (p=0.003), the relationship between colleagues (p=0.037), and the perception of pathology (p=0.001).

### Comparison Between Subgroups and Geographic Regions

Significant differences in satisfaction levels of workplace conditions, professional well-being, job satisfaction, and perception of pathology were observed between nonacademic junior, academic junior, non-academic senior, and academic senior pathologists (p<0.05) (Table 2).

**Table 2:** Comparison of satisfaction according to experience and academic status. The chi-square test was used to compare frequencies. The Kruskal-Wallis test was used in the comparison of >2 independent groups (the post-hoc Dunn test was also performed to better demonstrate the significant differences between 4 subgroups; please see supplement 7).

	**Non-academic junior pathologists** **n=115**	**Academic junior pathologists** **n=27**	**Non-academic senior pathologists** **n=84**	**Academic senior pathologists** **n=95**	**p**
**Median age**	33 (IQR: 3)	36 (IQR: 4)	45 (IQR: 9)	50 (IQR: 10)	**p<0.001**
**Gender, female/male**	89/26	24/3	64/21	65/29	p=0.19
Minnesota satisfaction questionnaire (mean score±SD*)	3.23±0.60	3.37±0.61	3.18±0.63	3.66±0.64	**p<0.001**
Provincial development level* ** 1** ** 2** ** 3** ** 4** ** 5** ** 6**	35% (n=40) 9% (n=10) 13% (n=15) 10% (n=12) 17% (n=20) 16% (n=18)	52% (n=14) 15% (n=4) 11% (n=3) 15% (n=4) 8% (n=2) 0% (n=0)	50% (n=42) 19% (n=16) 13% (n=11) 7% (n=6) 7% (n=6) 4% (n=3)	61% (n=58) 19% (n=18) 12% (n=11) 8% (n=8) 0% (n=0) 0% (n=0)	**p<0.001**
**Laboratory history** ** 0-2 years** ** 2-10 years** ** 11-20 years** ** >20 years**	6% (n=7) 34% (n=39) 34% (n=39) 32% (n=30)	0% (n=0) 22% (n=6) 30% (n=8) 48% (n=13)	5% (n=4) 18% (n=15) 38% (n=32) 39% (n=33)	0% (n=0) 13% (n=12) 14% (n=13) 74% (n=70)	**p<0.001**
**Total number of biopsies** ** 1-20.000** ** 20.001-40.000** ** >40.000**	62% (n=71) 32% (n=37) 6% (n=7)	15% (n=4) 59% (n=16) 26% (n=7)	48% (n=40) 37% (n=31) 15% (n=13)	27% (n=26) 45% (n=43) 27% (n=26)	**p<0.001**
**Number of pathologists** ** 1-5** ** 6-10** ** 11-15** ** >15**	57% (n=65) 33% (n=38) 6% (n=7) 4% (n=5)	37% (n=10) 30% (n=8) 22% (n=6) 11% (n=3)	57% (n=48) 27% (n=23) 12% (n=10) 4% (n=3)	22% (n=21) 40% (n=38) 17% (n=16) 21% (n=20)	**p<0.001**
**Subspecialization**	9% (n=10)	56% (n=15)	18% (n=15)	75% (n=71)	**p<0.001**
**Intra-departmental consultation**	90% (n=104)	100% (n=27)	89% (n=76)	97% (n=91)	p=0.07
**Conflicts with colleagues**	13% (n=15)	41% (n=11)	35% (n=30)	35% (n=33)	**p<0.001**
**Training satisfaction mean score±SD**	3.10±0.72	3.34±0.72	2.88±0.76	3.38±0.61	**p<0.001**
**Physical conditions satisfaction mean score±SD**	3.13±0.76	3.32±0.94	3.36±0.76	3.73±0.75	**p<0.001**
**Satisfaction with working conditions mean score±SD**	3.01±0.47	3.16±0.29	3.10±0.42	3.27±0.28	**p<0.001**
**Relationship with colleagues mean score±SD**	3.34±0.54	3.19±0.63	3.24±0.65	3.38±0.58	p=0.28
**Relationship with the staff mean score±SD**	3.55±0.49	3.55±0.37	3.46±0.54	3.59±0.42	p=0.55
**Perception of pathology mean score±SD**	3.40±0.53	3.81±0.38	3.54±0.57	3.85±0.49	**p<0.001**
**Satisfaction with monthly income (%)**	27% (n=31)	48% (n=13)	18% (n=15)	34% (n=32)	**p=0.004**
**Satisfaction with additional payments (%)**	13% (n=15)	19% (n=5)	14% (n=12)	20% (n=19)	p=0.12
**Being content for choosing pathology (%)**	81% (n=93)	93% (n=25)	79% (n=66)	83% (n=79)	p=0.21

**Junior:** <40 years old; senior ≥40 years old (16). SD: Standard deviation. IQR: Interquartile range.

*: Provincial development level is determined by the Ministry of Development based on socio-economic conditions (14).

**Figure 3:**
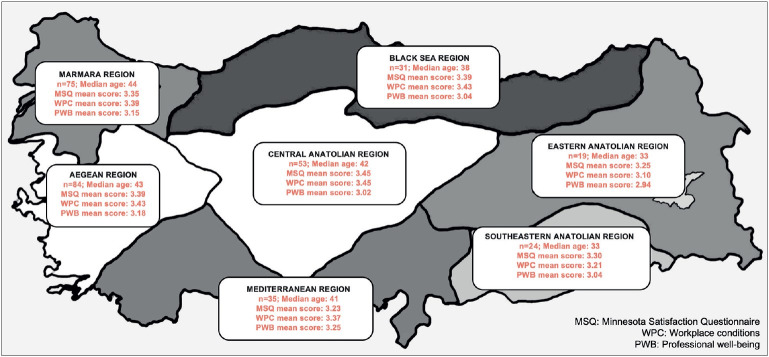
Comparison between the geographical regions. Respondents from East and South East Regions tended to be younger. Technical background was significantly better in the Marmara region, compared to the Black Sea and South East regions (not shown), while there was no significant difference regarding the Minnesota Satisfaction Questionnaire (MSQ), workplace condition (WPC) and professional well-being (PWB) scores.

Respondents from the East and South East Regions tended to be younger (p<0.001) (Figure 3). The experience of the laboratory, the number of total biopsies and pathologists, and the satisfaction level with the technical and physical background were significantly higher in the Aegean and Marmara Regions (p<0.05).

## DISCUSSION

This study, to the best of our knowledge, is the most detailed study investigating surgical pathologists’ job satisfaction and working conditions in Turkey, and also one of the few relevant studies in the English literature (4–6). Our results show that job satisfaction in pathology is basically influenced by both personal and environmental factors, such as in other professions.

### Academic pathologists and senior pathologists with ≥20 years of experience are more satisfied with their jobs and their working conditions

This is in fact consistent with findings of some other job satisfaction surveys among non-pathology subspecialties (18,19) and, more strikingly, academic pathologists have been reported to express better job satisfaction in another study by Jenkins et al. (4).

Age cannot be the only explanatory factor for the association between seniority and job satisfaction since age is not considered as a viable predictor of job satisfaction (20). However, younger individuals’ motivation has been shown to increase as they are offered more career opportunities (21) and, in this study, many non-academic junior pathologists stated that the institution they worked at was not their preferred choice (partly due to compulsory service), suggesting that loss of motivation can indeed be a contributing factor to their dissatisfaction. This may also indicate that senior pathologists are more satisfied because they have reached their career goals or that they just got accustomed to the situation and gave up hoping for better. However, further investigation is required to fully explore this aspect.

### Job satisfaction of pathologists is correlated with physical conditions of the pathology laboratory

This is somehow expected and also one of the main reasons why academic pathologists, senior pathologists, and pathologists working at larger laboratories expressed higher job satisfaction since academic institutions and large institutions usually have better physical background. On the other hand, half of the respondents were not satisfied with the technical and physical background of their laboratory and described insufficient and poor physical conditions in the grossing room and suboptimal conditions for microscopic examination, and many also complained about inexperienced and/or unknowing and reluctant staff/technicians. Moreover, 11% of the respondents had to set-up the pathology laboratory when they were first assigned to the hospitals they currently work at. Therefore, it is clear that workplace conditions are significantly heterogeneous in Turkey and the number of the pathologists who work at non-teaching hospitals is too high to be ignored since 87% of all the hospitals in Turkey are non-teaching hospitals (17,22). However, it may not be possible and/or rational to reserve the same amount of funding for every laboratory across the country. Thus, centralization of the pathology services, i.e. adopting a centralized pathology laboratory approach by determination of the need for a pathology laboratory by the number of the hospital beds and/or type and number of the surgical procedures in a hospital appear to be the best option to overcome the mentioned obstacles (23,24), and to improve surgical pathologists’ job satisfaction.

### The city/area where the pathologist lives in affects job satisfaction

Living in an advanced city or a developed area may offer both better job opportunities and better sociocultural activities, improving an individual’s quality of life. Although it is impossible to provide equal conditions for every pathologist, centralization of the pathology services may produce a solution for this problem as well.

### Shortfalls in the training system and pathology service design negatively affect junior pathologists’ motivation

Although we did not aim to assess training in pathology in detail, respondents declared that administration/management strategies, laboratory techniques, and molecular pathology as the weakest aspects of their residency programs. Working at laboratories with poor lab conditions contribute to junior pathologists’ dissatisfaction and a feeling of insecurity. Unfortunately, one fifth reported a fear of responsibility/lack of confidence to sign-out cases and another one fifth reported inexperience/lack of knowledge on several topics of pathology when they first started practicing. Hence, residency core curriculums must be standardized and updated to strengthen these aspects. Also, reporting of the complex cases should be encouraged in the residency period to overcome confidence problems. Training on hematopathology, dermatopathology and soft tissue/bone pathology should also be supported by special courses and by online training, given that these are the top 3 topics creating the need for a second opinion. Inter-institutional rotations or training of the trainers have been suggested as other solutions (24). Additionally, constructing a new training system focusing on subspecialty training may be of help and encourage pathology residents to specialize in areas that they are interested in. However, one should also consider that this will also prolong the training period and may cause a deterioration of the knowledge on surgical pathology. A thorough targeted study comparing training conditions and satisfaction in different institutions may help to describe permanent solutions in detail.

It should be noted here that, albeit low, a number of pathologists (4%) expressed their discontent with the current inter-institutional consultation mechanism and pointed to a main shortfall: lack of an official feed-back loop to follow-up the results and/or discuss opinions. Usubutun et al. reported that suspecting the diagnosis, need for immunohistochemical studies, patient requests, and need for molecular studies are the most frequent reasons for consultation in Turkey (25). The authors have also found that 59% of the consultant pathologists report the results to the first pathologist only if he/she asks for it (25). Therefore, official/written protocols are needed to enable institutional feedback, or telepathology options with digital whole slide imaging can be encouraged as a good option for consultation (26), also allowing online discussion and feedback, thus enhancing self-confidence which may improve job satisfaction.

### Interpersonal conflicts overshadow professionalism

Some respondents (mostly academic junior pathologists and pathologists who work at large institutions) expressed conflicts with their colleagues, mainly due to personal disagreements, subspecialization and caseload distribution, which may be attributable to the competition in larger settings that decreases job satisfaction in return. Interpersonal conflicts can be mediated by mutual compromise at a personal level, by adopting the concept of “team work”, or by administrative measures such as training (24). Although Güner et al. have claimed that Eastern cultures rely on apprenticeship rather than standardized programs in higher education (27), our professional organization(s) can take a leadership role to help departments and training programs define professionalism, ethical values, and codes of conduct for the practicing pathologist as suggested by Domen (28) and the concept of professionalism may also be implemented in standard training curriculums (29).

### Alarm signal: Burn-out is not infrequent among pathologists

Chronic fatigue due to excessive work load, sleep deprivation or decreased personal time, perceived threats such as making mistakes, loss of autonomy, inefficiencies in administrative tasks, balancing needs (multitasking), chronic stress due to workplace setting and personal factors have been suggested as factors that may contribute to burn-out symptoms among physicians (30). In our study, 21% expressed feeling pressured by the physicians for more rapid sign-out, which is an important factor that causes job-related stress. Almost half (45%) of the respondents reported to experience the feeling of being burnt-out and 69% described fatigue due to excessive working which is remarkably higher than the series of Fritzsche et al, in which depression and burn-out have been reported to affect 8% of pathologists (31). On the other hand, in a recent study on pathologists’ burn-out in the United States, 71% of the respondents expressed having felt burn-out at some time (32), and in another recent study among pathology residents and fellows, the majority also appraised their work-life balance as poor or fair (8). A previous study suggested that the main problem leading to unbalanced workload in pathology laboratories in Turkey to be the unequal institutional workload distribution (33). In other words, the workload in teaching hospitals is remarkably higher than in smaller non-teaching hospitals, and academic pathologists are expected to fulfill more daily tasks (routine sign-out, teaching, research etc.) (33). Ironically, the so-called “flexible work hours” seems to be translated as “excessive work hours” in academic settings. Moreover, the number of the pathologists in Turkey is also clearly below the European average (17,34). Therefore, strategic personnel planning is required to improve the quality and productivity, and increasing the number of the staff and pathologists in teaching hospitals should be considered. Adopting individual and/or institutional system-wide solutions to cope with burn-out are recommended (35).

### Back pain and neck pain are the most common work-related physical symptoms

In total, 65% of the respondents complained about musculoskeletal problems such as back and neck pain. Musculoskeletal problems have mostly been reported in the neck, shoulder and upper back areas, occasionally referred to as “pathologist’s hump” (31). Considering that these symptoms cause loss of manpower and fatigue, ergonomic workplace optimization is recommended (31).

### Pathologists are generally happy with their decision to become a pathologist, but they feel invisible to the public and other physicians

Despite the fact that pathology was not the first career choice of the majority and they chose pathology without actually knowing what pathologists do, more than 80% seem to be content with their choice. We have recently shown that flexible working conditions, scientific excitement, and attributed importance of pathology were the main reasons why pathology residents from different European countries chose pathology as a profession (7), and the findings of this study confirm that these are the most liked aspects of being a pathologist as well.

However, almost every respondent agreed that other physicians and patients do not actually know what pathology is and what pathologists do. Most respondents expressed discontent about the lack of reputation in public and underestimation of pathology by other physicians. They acknowledged that many physicians do not know the limitations and difficulties of the pathologic examination and the patients consider pathology as “*a laboratory test performed on automated machines*”. While the “invisibility” of pathology is not country-specific (7), pathologists who work at large laboratories and academic centers seem to have a more favorable perception of pathology, probably due to the positive impact of long-term collaboration, and establishing a “common language” with clinicians increasing the feeling of “being understood”. As it is quite difficult to publicize pathology and to establish significant co-operation with physicians in small hospitals, we think that a national effort led by professional organization(s) would be the best way to introduce pathology to the public, and more joint meetings are required to develop co-operation with other physicians.

### Pathologists feel discouraged by inadequate financial compensation

Only 20% of the respondents were satisfied with their wage, a very low number compared to the United States, where 63% of the pathologists feel satisfied with their income (6). As some of the respondents articulated, this may be attributed to the underestimation of several time-consuming procedures in pathologic examinations (gross examination, reporting of major resections etc.) and relative ignorance of the public, other physicians, and hospital administrations about the pathology workflow which results in less payment compared to other specialties. As might be expected, this leads to disappointment and frustration among pathologists and negatively affects their work motivation. At this point, the expectation from the professional organizations is simply “*to build awareness of the presence of many resentful and unhappy pathologists*” and to take steps to improve pathologists’ income.

### Annual attendance to pathology congresses/meetings is lower than desired and academic pathologists constitute the core population

The number of respondents who annually attended the national and international pathology meetings was very low (28%), especially among non-academic seniors (12% and 0%, respectively). The scientific content and financial status/support appear as the main determinants of attendance. Therefore, improvement of these factors may encourage more pathologists to attend the meetings, and increase professional excitement and job satisfaction.

### Gender seems to affect job satisfaction

Although this finding is somehow biased due to the large number of female respondents, female respondents were more satisfied with their working conditions and expressed better relationship with colleagues. Previously, it has been controversially claimed that women’s expectations are lower than men’s (36) and that women usually feel more satisfied with their jobs even when they have worse job options (36,37), which may also be true for pathologists. We, interestingly, found that male participants were more satisfied with their training, and felt more confident and prepared to sign out cases when they first started practicing. While self-esteem widely differs due to cultural differences (38), similar results were observed among nursing students as well (39). Whether this could actually be the case for pathologists needs further research.

The major limitations of our study were the relatively low response rate and the survey length. The survey was composed of 59 questions in addition to 20-item MSQ and this may have intimidated the potential respondents. However, the number of the respondents is within the 95%-97% confidence interval and response to online surveys tends to be low in general. In addition, despite its length, it was completed by 321 respondents from all geographic regions and all but 8 cities of Turkey, providing quite a panorama about the job satisfaction throughout the country.

To summarize; our results show that the factors comprising job satisfaction varies in different groups of pathologists. **Non-academic junior pathologists** are unhappy, feel more insecure as they more frequently work in smaller cities, and in less well-equipped laboratories. **Academic juniors** suffer from feeling burnt-out due to excessive and unbalanced workload, multitasking, and interpersonal conflicts**. Non-academic seniors** experience less conflict, are happy with working hours but are usually less satisfied with their monthly income and feel less motivated for participating in scientific meetings. **Academic seniors**, work in technically more advanced laboratories and express higher job satisfaction, but complain about the workload and multitasking.

In conclusion; despite all the discouraging conditions described in this study and their feeling of “invisibility”, most pathologists in Turkey are satisfied with their career choice, mainly because of their dedication to the importance of pathology for patient management. However serious improvements and innovation in the current system are necessary to overcome the feeling of burn-out and especially to foster junior pathologists’ hope and work motivation, and, therefore, to be able to offer a better future both to the pathologists and the patients.

## CONFLICT of INTEREST

The authors declare they have no conflict of interest.

## FUNDING

No funding was received.
